# Bacterial Profile and Antibiotic Resistance of ESKAPEE Pathogens Isolated in Intensive Care Units from Blood Cultures: A Cross-Sectional Study from Abu Dhabi, United Arab Emirates (2018–2022)

**DOI:** 10.3390/antibiotics14111142

**Published:** 2025-11-11

**Authors:** Ayesha Abdulla Al Marzooqi, Maryam Mohammed Bashir, Mohammed Ahmed Khogali, Abubaker Suliman, Collins Timire, Farida Ismail Al Hosani, Faisal Musleh Al Ahbabi

**Affiliations:** 1 Communicable Disease Department, Infectious Diseases Sector, Abu Dhabi Public Health Center (ADPHC), Abu Dhabi P.O. Box 5674, United Arab Emirates; 2 Institute of Public Health (IPH), College of Medicine and Health Sciences, United Arab Emirates University, Al Ain P.O. Box 15551, United Arab Emirates; m.bashir@uaeu.ac.ae (M.M.B.); ahmedm@uaeu.ac.ae (M.A.K.); ababaker.suliman@uaeu.ac.ae (A.S.); 3 International Union Against Tuberculosis and Lung Diseases, 75006 Paris, France; 4 The Global Institute for Disease Elimination (GLIDE), Abu Dhabi P.O. Box 111999, United Arab Emirates

**Keywords:** AMR, antibiotic resistance, ESKAPEE, ICU, blood infections, SORT IT, UAE

## Abstract

**Background:** Antibiotic resistance is a significant health problem in healthcare settings, especially intensive care units (ICUs), where patients are critically ill. This study aims to identify the bacterial profile and antibiotic resistance patterns of *Enterococcus faecium*, *Staphylococcus aureus*, *Klebsiella pneumoniae*, *Acinetobacter baumannii*, *Pseudomonas aeruginosa*, *Enterobacter*, and *Escherichia coli* (ESKAPEE) in blood specimens collected from adult patients admitted to the ICUs of public hospitals in Abu Dhabi, United Arab Emirates. The World Health Organization lists these pathogens as priority pathogens that greatly threaten humans. **Methods:** This cross-sectional study used routinely collected data through the AMR surveillance system between 2018 and 2022. **Results:** A total of 838 culture-positive blood specimens were reported during the study period, and 965 ESKAPEE pathogens were isolated. The most frequently isolated bacteria were *Klebsiella pneumoniae* (31%), *Escherichia coli* (22%), and *Staphylococcus aureus* (20%). *Acinetobacter baumannii* exhibited high resistance to Amikacin (81%), Meropenem (72%), and Imipenem (87%). *Escherichia coli* demonstrated resistance to Imipenem (42%) and Cefotaxime (54%). *Klebsiella* pneumoniae showed resistance to Imipenem (37%) and Cefotaxime (39%). *Staphylococcus aureus* showed resistance to Penicillin G (80%), Oxacillin (4%), and Ciprofloxacin (54%). **Conclusions:** The study showed a high prevalence of resistance in the most frequently isolated ESKAPEE pathogens in adult ICU patients. This brings into focus the need for appropriate infection control measures and strong antibiotic stewardship programs. The findings of the study support the ongoing efforts to deploy a better diagnostic tool for rapid pathogen identification, which is key in the targeted management of patients with bloodstream infection, especially in ICUs.

## 1. Introduction

Antibiotics are essential drugs for the treatment and prevention of bacterial infections [1]. However, the emergence and spread of antibiotic resistance undermine their effectiveness, leading to substantial morbidity, mortality, and economic implications worldwide [2,3].

The growing challenge of antibiotic resistance is especially concerning in intensive care units (ICUs) due to the many risk factors for hospital-acquired infections (HAIs) in these settings [4,5]. In addition, antibiotic resistance in ICUs limits treatment options, compromising patients’ survival, particularly in patients with bloodstream infections (BSIs), which are caused by bacteria in more than 90% of cases [6]. Thus, rapid detection of resistant bacteria in the bloodstream is vital to patient care because of the high mortality rates associated with BSIs [7].

The most common bacteria identified in BSIs were *Enterococcus faecium*, *Staphylococcus aureus*, *Klebsiella pneumoniae*, *Acinetobacter baumannii*, *Pseudomonas aeruginosa*, *Enterobacter*, and *Escherichia coli*, collectively known as the ESKAPEE pathogens. This group of multidrug-resistant bacteria is clinically difficult to treat and contributes to increased mortality rates attributable to antibiotic resistance [8]. They are also included in the World Health Organization (WHO) list of AMR priority pathogens for investment, surveillance, research, and development [2]. In 2021, the ESKAPEE pathogens were reported to be responsible for almost 75% of all deaths associated with antibiotic-resistant bacterial infections [8]. This alarming rate highlights the importance of adequate surveillance of the ESKAPEE pathogens, both at global and local levels.

The World Health Organization (WHO) recommends establishing antimicrobial stewardship (AMS) programs—a set of coherent and coordinated evidence-based interventions designed to promote appropriate and responsible use of antibiotics at both national and healthcare facility levels [9]. AMS reduces healthcare-associated infections (HAIs), curbs AMR, optimizes antimicrobial use, improves patient outcomes, and lowers healthcare costs. As such, AMS is considered a cornerstone in tackling antibiotic resistance in ICUs [10]. Effective monitoring and evaluation of these programs require robust information on the type of bacterial infections with resistant organisms to guide the selection of effective antibiotics for empirical treatment [1]. Moreover, conducting comprehensive and effective surveillance is crucial to track resistance patterns and identify potential factors influencing them [9].

To this end, the United Arab Emirates (UAE) has established a laboratory-based AMR sentinel surveillance system, encompassing a network of 35 public health facilities. Previous studies in the UAE over the past decade were limited to a few pathogens and selected hospitals [3,11,12]. Therefore, there is a need for a comprehensive assessment to provide insights into bacterial profiles and antibiotic patterns, particularly in ICU settings. Such information will provide the basis for evidence-based local and national stewardship programs and overarching AMR strategies [3].

This study aimed to identify the bacterial profile and antibiotic resistance patterns of ESKAPEE pathogens among culture-positive blood specimens obtained from adult patients admitted to the ICUs of public hospitals in the Emirate of Abu Dhabi, the UAE, from 2018 to 2022. The specific objectives were to report on (i) the distribution of blood culture positives by sociodemographic characteristics of the patients; (ii) the number and proportion of culture-positive bacterial isolates per year; (iii) their resistance patterns to selected antibiotics; and (iv) trends in the resistance of selected bacteria to specific antibiotics according to the WHO priority list.

## 2. Methods

### 2.1. Study Design

This was a cross-sectional study conducted using routinely collected, aggregated data from a laboratory-based AMR surveillance system in the Emirate of Abu Dhabi. United Arab Emirates, during the period from 2018 to 2022.

### 2.2. Study Setting

#### 2.2.1. General Setting

The Emirate of Abu Dhabi consists of three regions: Abu Dhabi, Al Ain, and Al Dhafra. Healthcare services are provided through a network of hospitals, primary healthcare centers, and specialized health facilities regulated by the Abu Dhabi Department of Health (DOH). Of the 64 hospitals in the Emirate, 35 participate in the antimicrobial resistance (AMR) surveillance. Eight of these hospitals have ICUs served by seven central laboratories that have adopted the AMR Surveillance System.

#### 2.2.2. Specific Setting

The laboratory-based surveillance system comprises a network of microbiology laboratories across the Emirate of Abu Dhabi, providing microbiology services for AMR surveillance sites since 2015. AMR phenotypical data are extracted by focal points at each participating surveillance site from the hospital/laboratory information systems (HIS/LIS) and reported regularly to the communicable disease department of the Abu Dhabi Public Health Center (ADPHC). The reported data include microbiological, clinical, and demographic information.

### 2.3. Study Population

Our study population included all blood culture-positive specimens for ESKAPEE pathogens obtained from adult patients (≥18 years) admitted to the ICUs of public hospitals in Abu Dhabi Emirate and served by laboratories participating in the AMR surveillance system from 2018 to 2022.

### 2.4. Collection of Blood Specimens in the ICUs

Blood specimens for culture and sensitivity were collected from all ICU patients with fever, hypothermia, or suspected infections using standard aseptic techniques, following the healthcare facility’s policy designed by the infection prevention and control team. Specimens were transported to laboratories participating in the AMR surveillance within 24 h.

### 2.5. Laboratory Processing, Bacteriological Culture, and Susceptibility Testing

Blood specimens collected for culture were processed in laboratories following the American Society for Microbiology guidelines [13]. Identification of bacteria and antimicrobial susceptibility testing (AST) were performed by medical professionals using at least one commercial automated system, including VITEK^®^ (BioMérieux, Marcy-l’Étoile, France), BD Phoenix™ (Becton Dickinson, Franklin Lakes, NJ, USA) and MicroScan (Beckman Coulter, Brea, CA, USA). AST was performed according to Clinical and Laboratory Standards Institute (CLSI) guidelines. If CLSI lacked breakpoints for certain pathogen–antibiotic combinations, alternative guidelines, such as EUCAST guidelines, were used. Results were recorded in laboratory information systems (LISs), with antibiotics commonly prescribed for Gram-positive and Gram-negative bacteria included in testing. Isolates were classified based on susceptibility to antibiotics, ensuring quality control through internal testing and external quality assurance schemes (EQASs), including College of American Pathologists Proficiency Testing (CAP Pt), American College of Physicians Medical Laboratory Evaluation (ACP-MLE), Joint Commission International (JCI), or Regional External Quality Assessment Scheme (REQAS). All laboratories were headed by licensed clinical pathologists or microbiologists, ensuring adherence to standards and accurate interpretation of results.

### 2.6. Data Extraction and Variables

Data on positive cultures and AMR for all eligible isolates were extracted from the electronic database of the AMR surveillance system. Extracted variables included the date the specimen was sent to a laboratory, age, gender, nationality, region, culture growth, bacteria isolated, and antibiotic resistance pattern (susceptible/resistant) to selected antibiotics. Bacterial isolates included in this study include the ESKAPEE pathogens (*Enterococcus faecium*, *Staphylococcus aureus*, *Klebsiella pneumoniae*, *Acinetobacter baumannii*, *Pseudomonas aeruginosa*, *Enterobacter*, and *Escherichia coli*).

The standard antibiotics selected for resistance testing among the Gram-negative bacteria included Ampicillin (AMP), Amikacin (AMK), Gentamicin (GEN), Tobramycin (TOB), Amoxicillin/Clavulanic acid (AMC), Piperacillin/Tazobactam (TZP), Ertapenem (ETP), Meropenem (MEM), Imipenem (IMP), Cefepime (FEP), Cefotaxime (CTX), Ceftazidime (CAZ), Ciprofloxacin (CIP), Aztreonam (ATM), Tetracycline (TCY), and Trimethoprim–sulfamethoxazole (SXT). For Gram-positive bacteria, selected standard antibiotics included Gentamicin (GEN), High-Level Gentamicin (HLG), Amoxicillin/Clavulanic acid (AMC), Ciprofloxacin (CIP), Levofloxacin (LVX), Moxifloxacin (MFX), Teicoplanin (TEC), Vancomycin (VAN), Clindamycin (CLI), Erythromycin (ERY), Linezolid (LNZ), Penicillin G (PEN), Oxacillin (OXA), Tetracycline (TCY), and Trimethoprim–sulfamethoxazole (SXT).

These antibiotics were selected according to the recommended international guidelines, and they are used in routine testing and reporting by clinical microbiological laboratories in the UAE. The bacterial resistance to selected antibiotics was used for trend analysis according to the WHO priority list.

### 2.7. Data Management and Analysis

All data management and analyses were conducted using RStudio statistical software version 4.3.2. We summarized categorical data using frequencies with the corresponding proportions. The chi-square test was used to analyze the trend in isolate proportions, and a *p*-value of <0.05 was considered significant.

### 2.8. Ethical Approval

Ethical approvals were obtained from the Abu Dhabi Health Research and Technology Ethics Committee of the Department of Health, Abu Dhabi (DOH/ADHRTC/2024/1445) and the Ethics Advisory Group (EAG) of the International Union Against Tuberculosis and Lung Diseases, Paris, France.

## 3. Results

### 3.1. Distribution of Blood Culture Positives of ESKAPEE by Sociodemographic Characteristics of Patients

Table 1 illustrates the distribution of the blood culture-positive ESKAPEE pathogens by sociodemographic characteristics of adult patients admitted to the intensive care units (ICUs) in the Emirate of Abu Dhabi from 2018 to 2022. A total of 838 culture-positive blood specimens were reported during the study period. The majority of these positive cultures were reported among patients aged ≥65 years (51%), non-Emiratis (60%), male patients (62%), and patients from Abu Dhabi (68%).

### 3.2. Annual Distribution of Bacterial Profiles of the ESKAPEE Pathogens Isolated from Blood Specimens

Table 2 shows the annual distribution of ESKAPEE pathogens isolated from blood specimens. A total of 965 isolates were reported during the study period. The most common isolates across all years were *Klebsiella pneumoniae* (31%), *Escherichia coli* (22%) and *Staphylococcus aureus* (20%), with no significant trend observed over time. The proportion of *Acinetobacter baumannii* significantly decreased over the five-year period (*p*-value = 0.01).

### 3.3. Patterns of Antibiotic Resistance Among the ESKAPEE Pathogens Isolates

Table 3 describes the resistance patterns of Gram-negative ESKAPEE pathogen isolates to selected antibiotics. *Acinetobacter baumannii* showed high resistance to Aminoglycosides (81% to Amikacin, 65% to Gentamicin, 18% to Tobramycin) and Carbapenems (72% to meropenem and 78% to Imipenem). *Escherichia coli* exhibited resistance to Carbapenems (42% to Imipenem), Cephalosporins (42% to Cefepime and 54% to Cefotaxime), and Fluoroquinolones (60% to Ciprofloxacin). Similarly, *Klebsiella pneumoniae* showed resistance to Carbapenems (37% to Imipenem), Cephalosporins (37% to Cefepime and 39% to Cefotaxime), and Ciprofloxacin (34%). *Pseudomonas aeruginosa* showed resistance to Aminoglycosides (5% to Tobramycin), Carbapenems (27% to meropenem and 13% to Imipenem), β-lactamase inhibitors (13% to Piperacillin/Tazobactam), Cephalosporins (20% to Ceftazidime) and Fluoroquinolones (15% to Ciprofloxacin).

**Figure 1:**
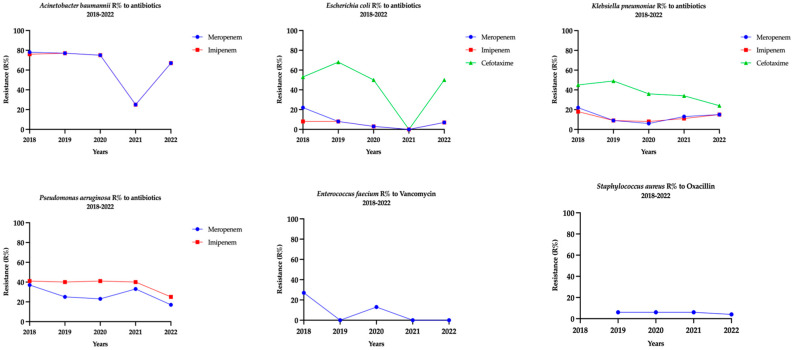
Trends in resistance of ESKAPEE bacteria to selected antibiotics in blood specimens of adults admitted to intensive care units of public hospitals included in AMR surveillance in Abu Dhabi Emirate, UAE (2018 to 2022).

Table 4 describes the resistance patterns among Gram-positive ESKAPEE isolates to selected antibiotics. *Staphylococcus aureus* demonstrated resistance to Aminoglycosides (2% to Gentamicin), Penicillin, and β-lactamase inhibitor combination (80% to Penicillin G and 4% to Oxacillin). It also showed resistance to Fluoroquinolones (54% to Ciprofloxacin, 36% Levofloxacin, and 35% to Moxifloxacin), Macrolides (32% to Erythromycin), and Tetracycline (10% to Tetracycline). *Enterococcus faecium* showed resistance to Aminoglycosides (65% to high-level Gentamicin), Macrolides (84% to Erythromycin), and Glycopeptides (9% to Vancomycin) (Figure 1).

## 4. Discussion

This study aimed to understand the bacterial profiles and resistance patterns of ESKAPEE pathogens in culture-positive blood specimens collected from adult patients admitted to the ICUs in the Emirates of Abu Dhabi, United Arab Emirates (UAE) over a five-year period. The study provided important information about the bacterial profile and resistance patterns among these priority pathogens, which have significant implications for the clinical management and survival of critically ill patients.

Our study showed that the majority of positive blood cultures were among elderly patients, non-Emiratis and those from the Emirate of Abu Dhabi. The higher proportion of culture positives in elderly patients may be due to their weak immune system, which increases their susceptibility to infections, and, often, the presence of comorbidities [14]. The observed high proportion of culture-positive specimens in non-Emirati male patients and those from Abu Dhabi can be explained by the high ratios of non-Emiratis to Emiratis and males to females in the UAE, along with Abu Dhabi being the biggest region in the UAE [15].

The most frequently isolated ESKAPEE pathogens, across all years, ranked in descending order were *Klebsiella pneumoniae*, *Escherichia coli*, and *Staphylococcus aureus*. Similar findings have been reported in other studies conducted in similar critical care settings [16,17]. This may be because these three pathogens are the leading causes of bloodstream infections (BSIs), especially in ICUs [6]. However, the UAE National AMR surveillance report showed that *Escherichia coli* was the most common among bacterial isolates across the country. This discrepancy with our study is probably because the national report contains data from different hospital units and reports on all types of specimens [18].

In terms of the resistance patterns among Gram-negative pathogens, *Acinetobacter baumannii* showed resistance to almost all classes of antibiotics, with alarmingly high resistance to Aminoglycosides (80% to Amikacin and 65% to Gentamicin) and Carbapenems (72% to Imipenem and 78% to Meropenem). Similar rates have been reported in other studies (Aminoglycosides up to 67%, Carbapenems up to 90%) [19,20,21]. A possible explanation for the Carbapenems resistance observed in our study can be attributed to the production of carbapenemase enzymes such as OXA-type variants (OXA-23 and OXA-24), which were reported in a study conducted across Gulf Cooperation Council (GCC) countries in 2018 [22]. Furthermore, a more recent study conducted in the Emirate of Dubai reported the detection of OXA-23, NDM-1, and GES-11 in *Acinetobacter baumannii* isolates [23]. These findings suggest the regional circulation of molecularly related resistant strains of *Acinetobacter baumannii* within healthcare settings in the region. The high resistance to both Aminoglycosides and Carbapenems makes *Acinetobacter baumannii*-related infections challenging to treat [24]. The resistance to Carbapenems is very worrisome, as these antibiotics are considered the last resort to treat infections caused by Gram-negative bacteria [25]. In ICU settings, the emergence of such resistance necessitates the need for effective infection control measures and the development of new treatment options, as patients in these units are severely ill and immunocompromised, and treatment with broad-spectrum antibiotics is crucial [26].

Similarly, *Escherichia coli* demonstrated high resistance to Carbapenems (42% to Imipenem), Cephalosporins (42% to Cefepime and 54% to Cefotaxime), and Fluoroquinolones (60% to Ciprofloxacin). Other studies on BSIs conducted in ICUs have reported similar resistance patterns of *Escherichia coli* to Cephalosporins (up to 95%) [19,27], Fluoroquinolones (up to 95%) [27,28] and Carbapenems (19% to 34%) [29]. Moreover, Carbapenems-resistant *Escherichia coli* has been steadily increasing in many parts of the world [30,31]. The high resistance patterns of *Escherichia coli* observed in our study can be attributed to the presence of the extended-spectrum-β-lactamase (ESBL) genes. A study by Alfaresi M et al, in the UAE identified several of these genes, including CTX-M, SHV, and TEM, which may have contributed to the high resistance rates observed [32]. These resistance patterns highlight the need for regular monitoring and enhanced stewardship programs. 

*Klebsiella pneumoniae* also showed resistance to Cephalosporins (39% to Cefotaxime), Carbapenems (37% to Imipenem), and Fluoroquinolones (34% to Ciprofloxacin). Thee resistance patterns *Klebsiella pneumoniae* align well with the literatures at regional [33] and global levels [34]. An analysis of the AMR surveillance data conducted in the UAE in 2021 has reported that up to 67.6% of *Klebsiella pneumoniae* isolates were resistant to Imipenem [18].

As for Gram-positive pathogens, *Staphylococcus aureus* demonstrated significant resistance to multiple antibiotics, including 80% to Penicillin G, 54% to Ciprofloxacin, 36% to Levofloxacin, and 32% to Erythromycin. The high resistance to Penicillin can be attributed to the widespread methicillin-resistant *Staphylococcus aureus* (MRSA), a leading cause of healthcare-associated infections in ICUs [35]. Of *Enterococcus faecium* isolates, 43% were resistant to Vancomycin. This is lower than the proportions (8.1%) reported at the national level [36] and in other countries in the region, such as Egypt (46%) [37]. However, high proportions (27–66%) of Vancomycin resistance in *Enterococcus faecium* were observed in several European countries [38,39].

Another notable finding in our study was the decreasing trend in the proportion of isolates over the years, which was significant for *Acinetobacter baumannii*. This aligns with the findings of the WHO global survey during the COVID-19 pandemic. The observed decrease in positive culture could be due to reduced surveillance of blood specimens, with respiratory specimens being prioritized during this period [40]. There is a need to reevaluate and enhance the efficiency of the surveillance system to effectively capture data for both routine monitoring and emergency situations. Another possible reason could be the implementation of exceptionally stringent infection prevention measures during the pandemic, which may have reduced infection transmission and, consequently, culture positivity rates, as demonstrated by an experimental study in Greece [41]. 

The strengths of this study include the relatively large sample size of culture-positive blood specimens, which allows for a comprehensive overview of the bacterial profiles and resistance patterns. Furthermore, the AMR surveillance system in Abu Dhabi covers all public hospital ICUs. The laboratory technicians in these hospitals are well-trained in processing and testing blood specimens to detect bacterial pathogens and follow a standardized protocol for performing antibiograms. In addition, we adhered to the STROBE guidelines for reporting epidemiological observational studies [42].

The study has some limitations. First, the surveillance system does not capture data on the total number of cultures performed, making it difficult to assess the trend of culture-positive blood specimens and the occurrence of blood infections. Second, only aggregate data were reported through the surveillance system, precluding a comprehensive understanding of factors associated with antibiotic resistance. Third, we did not investigate the genotypic mechanisms underlying high resistance exhibited by some bacterial pathogens to certain antibiotics, as this was beyond the scope of our study. However, we provided possible explanations based on findings of previous studies conducted in the region. Furthermore, due to the lack of clinical data, such as treatment outcomes, we were not able to evaluate the morbidity and mortality burden related to antibiotic resistance.

Despite the limitations, the study highlights important policy implications, which are fivefold. First, we recommend effective implementation of antibiotic AMR stewardship programs in hospitals to promote the rational use of antibiotics. Second, stringent infection control measures in ICUs should be enhanced to prevent cross-infections and the spread of resistant strains. Third, better diagnostic tools should be deployed to rapidly recognize resistance and personalize treatment based on susceptibility testing to ensure the most effective antibiotics are used, particularly in ICU settings where patients are critically ill. Further research can include evaluating the experience of implementing the Rapid PCR-based Blood Culture Identification Panel (BCID) test in one of the largest hospitals that participated in this study and, if found effective, scaling it up to other hospitals in the Emirate of Abu Dhabi. This would constitute a leap forward in enhancing detection of AMR, enabling earlier adjustment of therapy in critically ill patients, and improving treatment outcomes [43].

Fourth, the surveillance system must capture data on all cultures performed and link laboratory data with clinical data. Finally, the efficiency of the AMR surveillance in capturing both routine and emergency surveillance needs to be evaluated. Addressing these issues could reduce the burden of antibiotic-resistant pathogens in ICUs and strengthen AMR surveillance in the Emirate of Abu Dhabi.

## 5. Conclusions

The study showed a high prevalence of resistance in the most frequently isolated ESKAPEE pathogens in adult ICU patients. The resistance to Carbapenems is particularly concerning, as these antibiotics are the last option to treat Gram-negative infections. The study findings emphasize the need for effective infection control measures, robust AMR stewardship programs, and better diagnostic tools, especially in critical care settings. There is also a need to strengthen the AMR surveillance system in this setting.

## Figures and Tables

**Table 1:** Distribution of positive blood culture for ESKAPEE pathogens by sociodemographic characteristics of adult patients admitted to the intensive care units, Abu Dhabi Emirate, UAE (2018 to 2022, n = 838).

Characteristics	*n* (%)
Total ^¥^	838
Age Category (yrs)	
18–35	96 (12)
36–49	103 (12)
50–65	213 (25)
≥65	426 (51)
Gender	
Female	315 (38)
Male	523 (62)
Nationality	
Emirati	307 (37)
Non-Emirati	501 (60)
Unknown	30 (3)
Region	
Abu Dhabi	574 (68)
Al Ain	225 (27)
Al Dhafra	39 (5)

^¥^ Total number of patients with positive culture.

**Table 2:** Annual bacterial profiles of ESKAPEE isolates from blood specimens of adult patients admitted to intensive care units of public hospitals, Abu Dhabi Emirate, UAE (2018 to 2022).

Isolates	All Years *n* (%)	2018 *n* (%)	2019 *n* (%)	2020 *n* (%)	2021 *n* (%)	2022 *n* (%)	*p*-Value *
Total	965 (100)	275 (29)	216 (22)	187 (19)	143 (15)	144 (15)	
*Acinetobacter baumannii*	46 (5)	18 (7)	13 (6)	8 (4)	4 (3)	3 (2)	0.01
*Enterobacter* spp.	43 (4)	14 (5)	10 (5)	11 (6)	5 (3)	3 (2)	0.17
*Enterococcus faecium*	43 (4)	11 (4)	8 (4)	8 (4)	8 (6)	8 (6)	0.35
*Escherichia coli*	217 (22)	70 (25)	41 (19)	38 (20)	38 (27)	30 (21)	0.66
*Klebsiella pneumoniae*	297 (31)	84 (30)	59 (27)	66 (35)	47 (33)	41 (28)	0.82
*Pseudomonas aeruginosa*	132 (14)	35 (13)	36 (17)	22 (12)	15 (10)	24 (17)	0.88
*Staphylococcus aureus*	187 (20)	43(16)	49 (22)	34(19)	26 (18)	35 (24)	0.14

* Chi-Square test used for trend analysis. *p*-value is significant at <0.05.

**Table 3:** Antibiotic resistance patterns of Gram-negative bacterial isolates from blood specimens of adult patients admitted to intensive care units of public hospitals, Abu Dhabi Emirate, UAE (2018 to 2022).

		*Acinetobacter baumannii* *n* = 46		*Enterobacter* spp. *n* = 43		*Escherichia coli* *n* = 217		*Klebsiella pneumoniae* *n* = 297		*Pseudomonas aeruginosa* *n* = 132	
Class	Antibiotics	Tested N	R * (%)	Tested N	R (%)	Tested N	R (%)	Tested N	R (%)	Tested N	R (%)
Aminoglycosides	Amikacin	27	(81)	34	(0)	165	(1)	222	(7)	128	(4)
	Gentamicin	46	(65)	43	(5)	216	(21)	297	(16)	132	(5)
	Tobramycin	45	(58)	18	(0)	76	(28)	103	(28)	131	(5)
Pencillins	Ampicillin	-	-	-	-	217	(76)	-	-	-	-
Pencillins + β-lactamase inhibitor combination	Amoxicillin/Clavulanic acid	-	-	-	-	208	(38)	285	(36)	-	-
	Piperacillin/Tazobactam	46	(72)	43	(33)	217	(18)	297	(27)	128	(13)
Carbapenems	Ertapenem	-	-	31	(10)	164	(5)	214	(11)	-	-
	Meropenem	46	(72)	43	(5)	213	(5)	296	(13)	132	(27)
	Imipenem	36	(78)	30	(13)	149	(42)	206	(37)	127	(13)
Cephalosporins	Cefepime	36	(78)	30	(13)	149	(42)	206	(37)	127	(13)
	Cefotaxime	**-**	**-**	39	(31)	210	(54)	286	(39)	-	-
	Ceftazidime	46	(72)	-	-	-	-	-	-	132	(20)
Fluoroquinolones	Ciprofloxacin	46	(70)	43	(7)	217	(60)	296	(34)	132	(15)
Monobactams	Aztreonam	-	-	1	(0)	6	(100)	2	(100)	55	(18)
Folate pathway inhibitor	Trimethoprim–sulfamethoxazole	46	(50)	43	(10)	217	(55)	296	(30)	-	-
Tetracyclines	Tetracycline	12	(42)	14	(14)	69	(55)	92	(37)	-	-

* R—resistant; interpretation should be made with caution where fewer than 30 isolates were tested. (-) The results were not reported for antibiotics that are clinically ineffective according to CLSI guidelines.

**Table 4:** Antibiotic resistance patterns of Gram-positive bacterial isolates from blood specimens of adult patients admitted to intensive care units of public hospitals, Abu Dhabi Emirate, UAE (2018 to 2022).

		*Enterococcus faecium* *n* = 43		*Staphylococcus aureus* *n* = 187	
Class	Antibiotic	Tested N	R * (%)	Tested N	R (%)
Aminoglycosides	Gentamicin	-	-	122	(2)
	High-level Gentamicin	40	(65)	-	-
Pencillins + β-lactamase inhibitor combination	Amoxicillin/Clavulanic acid	-	-	6	(0)
	Penicillin G	-	-	122	(80)
	Oxacillin	-	-	122	(4)
Lincosamides	Clindamycin	-	-	122	(16)
Fluoroquinolones	Ciprofloxacin	10	(60)	11	(45)
	Levofloxacin	7	(71)	39	(36)
	Moxifloxacin	-	-	121	(35)
Macrolides	Erythromycin	43	(84)	122	(32)
Glycopeptides	Teicoplanin	36	(11)	-	-
	Vancomycin	43	(9)	-	-
Tetracyclines	Tetracycline	-	-	122	(10)
Folate pathway inhibitor	Trimethoprim–sulfamethoxazole	-	-	122	(15)
Oxazolidinones	Linezolid	43	(2)	122	(0)

* R—resistant; interpretation should be made with caution where fewer than 30 isolates were tested. (-) The results were not reported for antibiotics that are clinically ineffective according to CLSI guidelines.

## Data Availability

Restrictions apply to the datasets. The datasets presented in this article are not readily available because of information security and data privacy requirements. Requests to access the datasets should be directed to the Department of Health Abu Dhabi, Abu Dhabi Public Health Center.
